# Synergistic nano-bioorganic amendments enhance soil properties and microbial structure in coastal saline soils

**DOI:** 10.3389/fmicb.2026.1720097

**Published:** 2026-03-09

**Authors:** Meng Xiao, Cheng Chen, Rongjiang Yao, Xiuping Wang, Guangming Liu

**Affiliations:** 1 Yellow River Conservancy Technical University, Henan Small Watershed Ecological Water Conservancy Engineering Technology Research Center, Kaifeng, China; 2 State Key Laboratory of Soil and Sustainable Agriculture, Institute of Soil Science, Chinese Academy of Sciences, Nanjing, China; 3 Institutes of Coastal Agriculture, Hebei Academy of Agriculture and Forestry Sciences, Tangshan, China

**Keywords:** coastal saline-alkali farmland, driving factors, functional gene prediction, organic amendment, soil microbial structure

## Abstract

**Introduction:**

Soil salinization threatens global food security and sustainable land use. Ameliorating coastal saline soils with exogenous amendments is crucial. Bio-organic fertilizer (OF) and nano-carbon (NC) are promising green amendments, but their comparative and combined effects on soil properties and microbial communities are not fully understood.

**Methods:**

A field experiment was conducted in coastal saline soil (Ninghe District, Tianjin, China). Four treatments were established: control (CK, no amendment), OF application, NC application, and combined application of OF and NC (FC). Soil physicochemical properties and microbial community structure (via 16S and ITS rRNA gene sequencing) were analyzed.

**Results:**

The FC treatment most effectively improved soil properties, significantly reducing bulk density, pH, salinity, and sodium adsorption ratio (SAR), while increasing porosity, water content, and nutrient (N, P) availability. Soil bacterial diversity (Ace, Chao1, Shannon indices) increased significantly in all amendment treatments compared to CK, with the highest values in NC and FC treatments. Amendment application altered microbial community composition, enriching specific bacterial taxa (e.g., *Firmicutes, Desulfobacterota* in FC) and fungal taxa. Redundancy analysis identified soil salinity and pH as key drivers of bacterial community structure, whereas fungal communities showed a distinct, less correlated response pattern.

**Discussion:**

The synergistic application of nano-carbon and bio-organic fertilizer (FC) created a more favorable soil habitat, rapidly ameliorating physicochemical conditions which directionally shaped the bacterial community. Bacterial and fungal communities responded differently to amendments, suggesting divergent assembly mechanisms. The FC strategy demonstrates high potential for the initial restoration of saline-alkali soils by enhancing soil health primarily through rapid physicochemical improvement and modulation of the soil microbiome, particularly bacteria. Future work should focus on functional validation of predicted metabolic shifts and assessment of agronomic outcomes.

## 1 Introduction

Arable land is essential for global food production (Shi et al., 2021). However, soil salinization poses a severe threat to the sustainable use of cultivated land. Elevated soil salt concentrations induce osmotic and ionic stress, impairing plant photosynthesis, reducing agricultural productivity, and endangering ecological stability and human wellbeing (Yao et al., 2021; Kumar et al., 2022). According to the Food and Agriculture Organization (FAO, 2020), over 930 million hectares are currently affected by soil salinization, with this area expanding at an annual rate of 1.4% (Liu et al., 2022). Given these trends, soil reclamation has become a critical strategy for enhancing food production and supporting sustainable development (Miranda et al., 2021; Shi et al., 2021). Therefore, it is imperative to implement long-term, sustainable soil improvement practices, particularly in coastal regions where salinity is exacerbated by both natural and anthropogenic factors.

Extensive research have demonstrated that amending saline-alkali soils with exogenous materials is an effective remediation approach (Sun et al., 2019; Zhou et al., 2019; Kumar et al., 2022). Several established methods involve the development of soil amendments. For instance, the application of gypsum (CaSO_4_·2H_2_O) releases calcium ions (Ca^2+^), which helps displace sodium ions, encourages clay flocculation, and enhance soil structural stability (Amer et al., 2023; Shaaban et al., 2023). Additionally, amendments containing acidic functional groups can activate solidified calcium and magnesium ions through complexation, thereby facilitating cation exchange on soil colloids (Liu et al., 2022; Yao et al., 2021). Moreover, sulfuric and nitric acids lower soil pH via acid-base neutralization reactions (Farooqi et al., 2023). However, acidification may reduce soil pH excessively, potentially triggering aluminum toxicity and increasing osmotic stress in plants (Zhang et al., 2015; Zhou et al., 2019). Furthermore, traditional improvement materials may be inadequate for managing the ongoing salt accumulation and migration characteristic of secondary salinized soils, especially under deteriorating environmental conditions. Thus, there is growing interest in sustainable materials that not only mitigate salinity but also improve long-term soil fertility.

Bio-organic fertilizer, which serves as both a nutrient supplement and a fertilization-enhancing exogenous material, is widely utilized to boost soil available nutrients, restore physicochemical properties, and alleviate salinity stress (Li et al., 2023; Somenahally et al., 2023; Taher et al., 2023). Research indicates that bio-organic fertilizer can reduce soil salinity and osmotic stress by releasing mineral nutrients such as K^+^, Ca^2+^, and Mg^2+^, while also enhancing the cation exchange capacity (CEC; Abbas et al., 2022; Xiao et al., 2022; Han et al., 2023). Furthermore, nanoparticles, ranging from 1 to 100 nm, have been increasingly used in agriculture. Specifically, nanocarbon particles are noted for their solubility and extensive surface area, which support water and nutrient retention and improve fertilizer efficiency (Zhou and Chen, 2017; Cao et al., 2021; Gao et al., 2023). (Xiao et al. 2025) have revealed that nano-organic carbon has potential advantages in regulating water and salt movement on saline soil. However, the comparative effectiveness of nanocarbon as green amendments in improving saline soils against bio-organic fertilizers remains to be clarified.

Soil microorganisms play a pivotal role in nutrient cycling, soil quality enhancement, and ecosystem functioning, including processes such as organic matter decomposition (Cao et al., 2023; Wang X. et al., 2023; Wang Y. et al., 2023). Microbial communities, composed of bacteria and fungi, exhibit substantial variation in abundance and dominant taxa across different environments and ecosystems (Zhang et al., 2018). For instance, wetland soils are characterized by highly diverse bacterial communities dominated by *Proteobacteria, Chloroflexi, Acidobacteria*, and *Verrucomicrobia* (Wang X. et al., 2023). Notably, forest soils in southwest China harbor a higher abundance of Gram-negative (G–) bacteria compared to bare soils (Dong et al., 2023). The structure of soil microbial communities is influenced by several edaphic factors, including nutrient availability, hydrological conditions, and soil type (Gardner et al., 2023). Salinity stress exerts a significant influence on microbial community composition, with evidence indicating that certain communities can adapt to or tolerate saline conditions, particularly in chronically saline or alkaline environments (Edwards et al., 2023; Ni et al., 2023). Studies have shown that fungi may exhibit greater resilience under stress, whereas bacteria may be more effective in facilitating the restoration of saline-alkaline soils through direct metabolic activities or by promoting plant growth (Zhang et al., 2021). Despite extensive research on microbial abundance, adaptive traits, and dominant taxa in salt-affected soils, the dynamics of microbial community structure and the underlying restoration mechanisms in response to micro-environmental changes remain poorly understood.

In this study, we hypothesized that (1) the application of bio-organic fertilizer and nano-carbon amendments would alleviate the saline-alkali characteristics of coastal saline soil and enhance its physical and chemical properties; (2) these amendments would increase microbial community species diversity and alter community structure composition. Furthermore, this study aims to (1) characterize shifts in soil microbial communities in response to bio-organic fertilizer and nano-carbon additions in coastal saline soil and (2) investigate the driving factors and mechanisms of community structure changes in the study area following the introduction of exogenous materials. This research contributes to a deeper understanding of how innovative soil amendments, particularly in combination with bio-organic fertilizers, influence soil properties and microbial ecology in degraded saline environments.

## 2 Material and methods

### 2.1 Site description

The experiment was conducted on a farm located in Ninghe District of Tianjin, China (117°30′4^′′^E, 39°25′54^′′^N). The soil type of the study area is saline soils (McGeorge, 1954; Table 1). The climate in this area is characterized by a continental, warm-temperate, semi-arid, semi-humid monsoon climate. The average annual precipitation was 590 mm, occurring mainly in summer over 65 days. The average annual temperature was 12.2 °C, with the highest and lowest temperatures recorded in July (31 °C) and January (1.8 °C), respectively. Annual total evaporation was approximately 1,700 mm, while annual total sunshine duration was 2,400 h, with May having the longest days (8 h per day) and the most radiation. The average relative humidity peaked at nearly 80% in July and August.

**Table 1:** Basic physicochemical properties of soil profiles in the experimental area.

**Soil depth (cm)**	**pH**	**EC (μs cm^−1^)**	**Salt content (g kg^−1^)**	**Soil texture composition (%)**			**SOC (g kg^−1^)**	**AK (mg kg^−1^)**	**AP (mg kg^−1^)**
				**Sand (2.0–0.05 mm)**	**Silt (0.05–0.002 mm)**	**Clay (**<**0.002 mm)**			
0–20	8.78	634.20	2.26	19.04	33.78	47.18	7.29	210.67	16.05
20–40	8.88	598.00	2.03	19.30	31.60	49.10	6.85	193.00	16.18

Soil samples were collected from the experimental site in Ninghe District, Tianjin, China, during the pre-sowing period in April 2023. EC, SOC, AP, and AK represent soil electrical conductivity, organic carbon, available soil phosphorus, available soil potassium, respectively.

### 2.2 Materials and experimental design

Four treatments were established for this study, CK (no amendments added), OF (bio-organic fertilizer applied), NC (nano-carbon addition), and FC (bio-organic fertilizer and nano-carbon addition). The application rate of bio-organic fertilizer was 6,000 kg ha^−1^, and nano-carbon was applied at 150 kg ha^−1^. Organic material was uniformly mixed into the 0–20 cm soil layer 1 week before rice transplanting. Each treatment was replicated three times in separate experimental plots, each with an area of 1,320 m^2^ (24 m × 55 m), and separated by field ditches covered with plastic film, extending to a soil depth of 70 cm. The experiment began in March 2023 (the date of crop application of the amendment) and ended in November 2023 (the sampling date).

The bio-organic fertilizer used in this study, provided by Nanjing Ningliang Bio-Fertilizer Co., Ltd., comprised high-quality chicken manure and cow manure fully fermented and decomposed. The bio-organic fertilizer has a pH of 8.2, an electrical conductivity (EC) of 23.2 mS cm^−1^, and an organic matter content of 515 g kg^−1^. The total nitrogen content is 25.6 g kg^−1^, with P_2_O5 at 22.4 g kg^−1^ and K_2_O at 18.6 g kg^−1^. The viable microbial count is recorded at 2.5 × 108 CFU g^−1^, and the amino acid content is measured at 20 g kg^−1^. This fertilizer contains Streptomyces species, Bacillus genus bacteria, and *Aspergillus niger* fungi, developed by the Jiangsu Provincial Center for Organic Solid Waste Innovation (Xiao et al., 2025). The nano-carbon is produced through the electrolysis of double graphite electrode plates, resulting in nano-carbon particles with diameters ranging from 10 to 100 nm and a mass fraction (The mass percentage of nano-carbon materials relative to deionized water) of 3–5% (Xiao et al., 2025). This dispersion is devoid of nutrients such as nitrogen (N), phosphorus pentoxide (P_2_O_5_), and potassium oxide (K_2_O). The nano-carbon fertilizer used in this study has a pH value of 3.8, EC of 265.3 μS cm^−1^, and is provided by Nanjing Jiamachi Ecological Engineering Co., Ltd.

### 2.3 Sample collection and analysis

Samples were extracted from five randomly selected points within each plot and composited into one sample. After being gathered, 12 soil mixture samples were brought to the lab in sealed plastic bags. Half of each soil sample was stored at 4 °C for biological property analysis, while the other half was air-dried, crushed, and sieved through 1 and 0.15 mm sieves for laboratory analysis of soil chemical properties. Based on the Unified Soil Classification System (ASTM, 2011), the soil was classified as clay of high plasticity (CH), with a topsoil (0–20 cm layer) bulk density of 1.45 g cm^−3^. The average salt content ranged between 2 and 2.3 g kg^−1^.

Soil pH and EC were determined in a 1:5 (soil: distilled water) ratio using a pH meter (LE703, Mettler-Toledo, Shanghai) and an EC meter (LE438, Mettler-Toledo, Shanghai), respectively. Soil texture was determined using the hydrometer method (Chinese Standard, Type A) and classified according to the USDA soil texture classification system. Water-soluble salt content was measured using the mass method (Bao, 2000). Soil bulk density and soil porosity were determined via the core method (Bao, 2000). Water-soluble basic ions were determined by flame photometry (Bao, 2000). Soil moisture content was determined via the oven-drying method (Bao, 2000). Soil organic carbon (SOC) was measured using the external-heat potassium dichromate oxidation method (Bao, 2000). Soil-available nitrogen (AN) was determined via the alkali-hydrolyzed diffusion method, and available soil potassium (AK) was determined by flame photometry following extraction with sodium bicarbonate, while soil available phosphate (AP) was determined through the molybdenum antimony colorimetric method (Bao, 2000). Soil enzyme activities including soil alkaline phosphatase (S-AKP), urease (S-UE), and catalase (S-CAT) were extracted using enzyme activity kits and measured using a microplate reader (Epoch2, BioTek, USA), with kits provided by Beijing Solarbio Science & Technology Co., Ltd.

### 2.4 Soil microbial DNA extraction and high-throughput sequencing

Soil total DNA was extracted using the FastDNA^®^ SPIN Kit for Soil Microbiome (Norcross, MP, USA). DNA concentration and purity were assessed using Nanodrop2000 (Thermo Scientific, Wilmington, DE) and examined via electrophoresis on 1% agarose gel. Amplicons in the V3–V4 region were sequenced using 16s rRNA and ITS1F/ITS2R primers with barcode markers. Bacterial PCR primers were 338F (5′-ACTCCTACGGGAGGCAGCAG-3′) and 806R (5′-GGACTACHVGGGTWTCTA AT-3′). Fungal PCR primers were ITS1F (5′-CTTGGTCATTTAGAGGAAGTAA-3′) and ITS2R (5′-GCTGCGTTCTTCATCGA TGC-3′). PCR products were pooled after amplification, and library construction was performed before sequencing on the Illumina NovaSeq platform (Shanghai Meiji Biomedicine Technology Co., Ltd.). Quality control and sequence analysis were conducted using QIIME2, with denoising performed using DADA2/Deblur to obtain Amplicon Sequence Variant (ASV) representative sequences and abundance information.

### 2.5 Statistical analysis

Based on ASV analysis, alpha diversity indices including ACE, Chao1, Shannon, and Simpson were calculated using random sampling. Beta diversity was demonstrated using non-metric multidimensional scaling (NMDS) analysis based on Bray-Curtis distance. To test for significant differences in community composition among different treatment, Analysis of Similarities (ANOSIM) was conducted based on the Bray-Curtis dissimilarity matrix using the anosim function from the vegan package. The resulting global R statistic and its significance (*p*-value) are reported. Linear discriminant analysis Effect Size (LEfSe) was conducted using the online module of the “microeco” package in R to identify key microbial taxa that characterize differences between sample groups (LDA > 2.0, *p* < 0.05). Linear Discriminant Analysis (LDA) was conducted on standardized data to visualize group separation and identify key discriminatory variables. Pearson correlation analysis was employed to analyze the correlation between soil microbial communities and soil properties. Redundancy analysis (RDA) was performed to analyze the relationships between soil bacterial and fungal communities and soil chemical properties (class level). The relationship between community structure (Bray-Curtis distance) and environmental gradients (Euclidean distance of normalized environmental variables) was examined using a Mantel test via the mantel function in the vegan package.

Data analysis was carried out using Microsoft Excel 2019 (version 16.0.15629) and SPSS (version 20.0, IBM, Armonk, New York, USA), with one-way ANOVA followed by Duncan's new multiple-range test to determine treatment significance. Redundancy analysis was conducted using Canoco5 software (version 5.02), and figures were generated using Origin software (version 9.65.169, Origin Lab). Multivariate analyses (ANOSIM, Mantel test, LDA) and Pearson correlations were conducted in R to assess group differences, matrix correlations, variable relationships, and group separation, with visualizations created using ggplot2.

## 3 Results

### 3.1 Soil physicochemical characteristics

Significant differences were observed in soil bulk density (BD) and porosity (Sp) among the various treatments (*p* < 0.05; Table 1). The highest bulk density (1.44 g cm^−3^) was recorded in CK, while the lowest (1.27 g cm^−3^) was in FC. Conversely, CK treatment exhibited the lowest porosity, whereas the NC treatment had the highest. Compared to CK, soil pH, EC, and salt content were significantly reduced in the other treatments (*p* < 0.05). The highest SAR was observed in CK, and the SAR of FC was significantly decreased than CK. In addition, FC treatment was significantly increased soil water content (SWC). Soil organic carbon content differed significantly among various treatments (*p* < 0.05), ranging from 10.47 to 8.6 g kg^−1^. The treatments were ranked as NC > FC >OF > CK for both soil available nitrogen (AN) and available phosphorus (AP). Similarly, soil urease (S_UE) and alkaline phosphatase (S_AKP) activities followed the same trend as AN and AP among the different treatments. However, no significant differences were found between soil available potassium and catalase among different treatments.

### 3.2 Soil microbial community diversity

**Figure 1:**
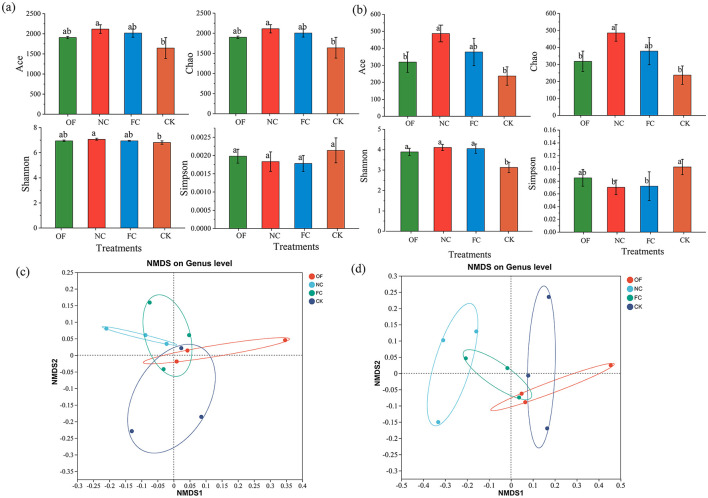
Alpha and beta diversity of soil microbial communities under different treatments. **(a)** Alpha diversity of soil bacterial communities; **(b)** alpha diversity of soil fungi communities. Indices include the observed richness (Ace and Chao1), Shannon diversity, and Simpson index. Different lowercase letters above the bars indicate statistically significant differences among treatments (OF, NC, FC, CK) according to Duncan's multiple range test (*p < 0.05). Values are presented as mean ± standard deviation (*n* = 3 independent experimental plots). Non-metric multidimensional scaling (NMDS) ordination of bacterial **(c)** and fungal **(d)** community structures based on Bray-Curtis dissimilarities. Ellipses represent the 95% confidence interval for each treatment group. OF, bio-organic fertilizer; NC, nano carbon; FC, bio-organic fertilizer combined with nano carbon; CK, no amendments addition. Different lowercase letters indicate significant differences between OF, NC, FC, and CK within the same stand (Duncan's multiple tests, *p* < 0.05). All data showed with mean value ± standard deviation (*n* = 3). Corresponding significance levels, *p < 0.05, **p < 0.01, ***p < 0.001.

There was a significant difference in soil bacterial diversity among the treatments (*p* < 0.05; Figures 1a, b). Compared to CK, the Ace and Chao1 diversity in the OF, NC, and FC treatments increased significantly by 16–28%. The NC treatment exhibited the highest Ace and Chao1 diversity values, indicating a greater richness of bacterial species. A similar trend was observed in the Shannon index across treatments. On the contrary, the Simpson index of the control group was the highest which indicates that the larger the index was, the lower the community diversity was, and the Simpson index was not significantly different among other treatments. Apart from the Simpson index, the Ace, Chao1, and Shannon indices generally followed the trend NC > FC > OF > CK (*p* < 0.05; Figure 1b). In contrast to bacterial diversity indices, fungal diversity indices showed considerable fluctuations among the different treatments.

NMDS analysis was utilized to illustrate the variance between various samples (Figures 1c, d). The difference among various samples was minimal, particularly in the NC treatment at the bacterial gene level. However, there was a more pronounced distinction between treatments NC and CK at the fungal gene level. Concurrently, samples derived from ostensibly identical treatments displayed noteworthy heterogeneity, manifesting in appreciable dissimilarities between them.

### 3.3 Microbial community composition and difference analysis

**Figure 2:**
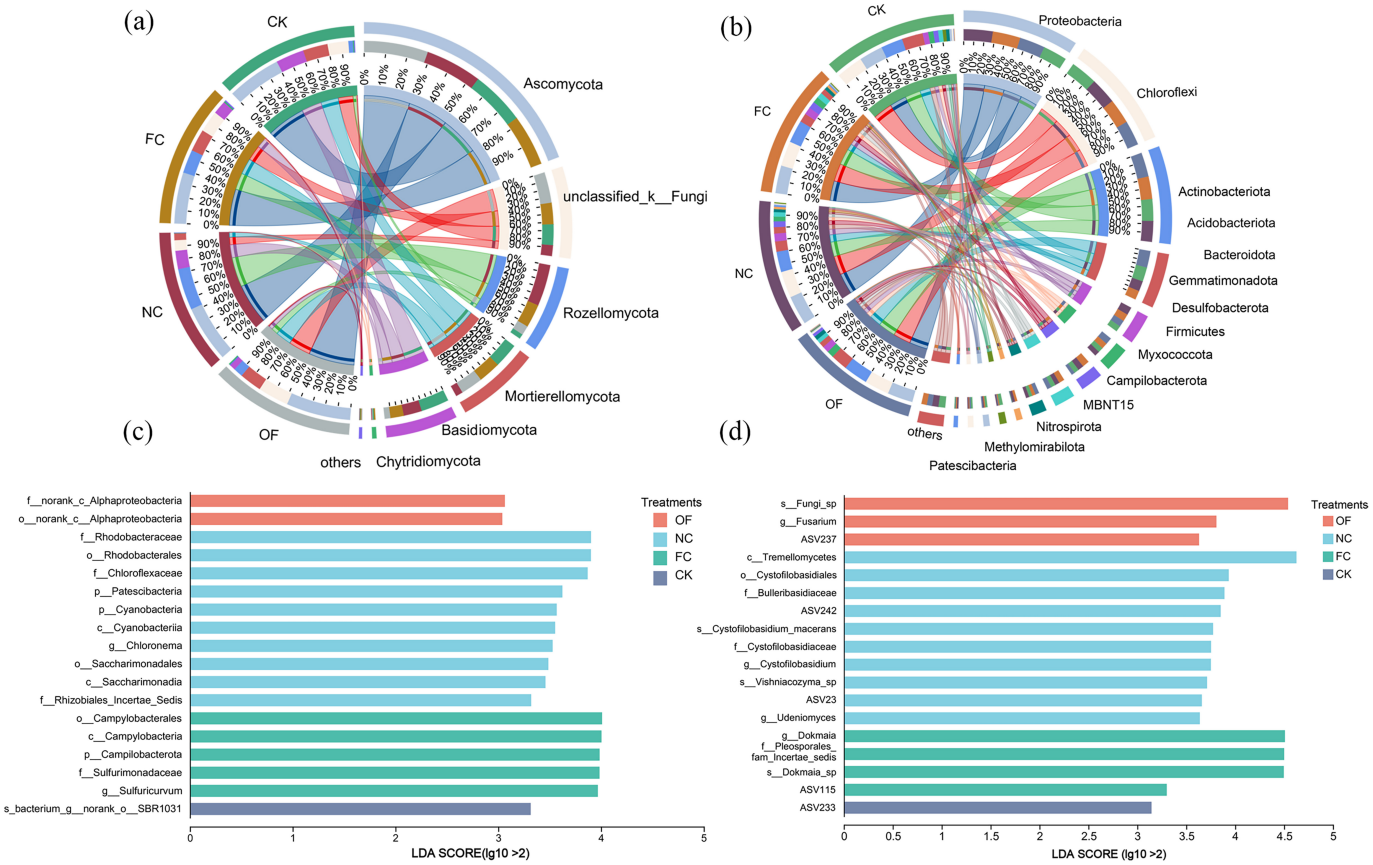
Soil microbial community structure between different treatments (only taxa with a relative abundance exceeding 1% are displayed). **(a)** Soil bacterial communities structure; **(b)** soil fungi communities structure. **(c)** Linear discriminant analysis (LDA) of soil bacterial; **(d)** linear discriminant analysis (LDA) of soil fungal communities. OF, bio-organic fertilizer; NC, nano carbon; FC, bio-organic fertilizer combined with nano carbon; CK, no amendments addition. Corresponding significance levels.

**Figure 3:**
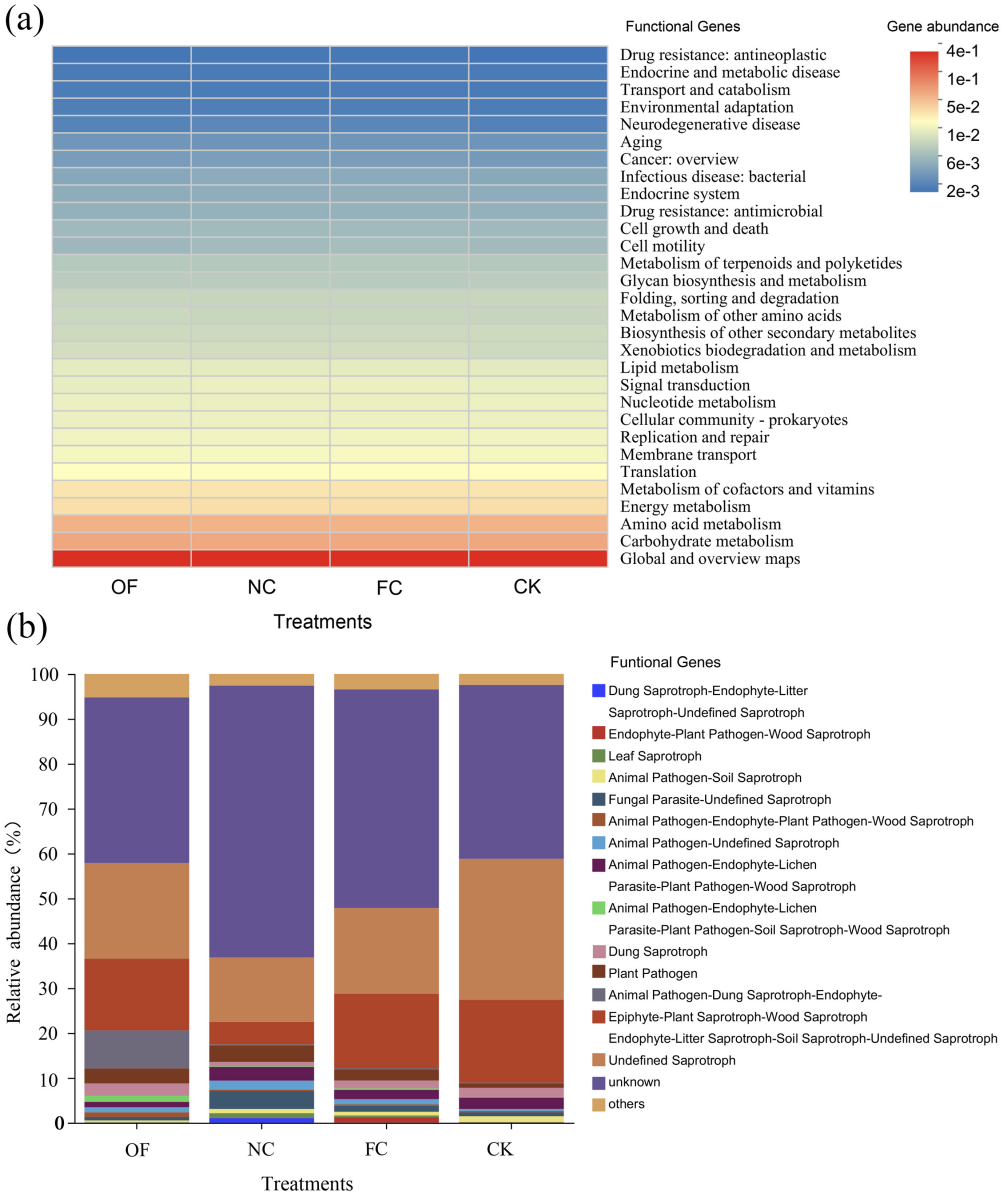
Prediction of functional genes in bacterial communities **(a)** and fungal communities **(b)** under different amendment treatments. OF, bio-organic fertilizer; NC, nano carbon; FC, bio-organic fertilizer combined with nano carbon; CK, no amendments addition. Corresponding significance levels.

At the bacterial phylum level, 14 bacterial phyla exhibited higher abundance, notably including *Proteobacteria, Chloroflexi, Actinobacteriota, Acidobacteriota, Bacteroidota, Gemmatimonadota, Desulfobacterota*, and *Firmicutes* as the predominant species (Figures 2a, b). Compared to CK, the FC treatment showed a higher abundance of *Desulfobacterota* and *Firmicutes*. At the fungal phylum level, 7 dominant species occupied prominent positions, such as *Ascomycota, Rozellomycota, Mortierellomycota, Basidiomycota*, and *Chytridiomycota* (Figure 3b). Particularly, *Mortierellomycota* ranged from 6 to 53% across different treatments. The FC treatment exhibited a higher (40%) abundance of *Chytridiomycota* compared to CK (28%).

Linear Discriminant Analysis (LDA) was utilized to elucidate distinctions among various treatments, showcasing variations in microbial taxa among samples (Figures 2c, d). *Proteobacteria* emerged as indicator bacteria for the OF treatment (Figure 2c). Notably, FC exhibited abundance in *Desulfobacteria* and *Desulfobacterota*, whereas CK showed enrichment in *Acidobacteriota*. LEfSe analysis identified multiple fungal taxa as discriminant biomarkers for the FC treatment (Figure 2d). The most prominent was an unclassified fungal species (s_Fungi_sp), followed by taxa within the *Cystofilobasidiales* order and the genus *Fusarium*. No significant fungal biomarkers were detected for the CK, NC, or OF treatments in this analysis.

### 3.4 Predicted functional gene analysis for microbial communities

Phylogenetic investigation of communities by reconstruction of unobserved states (PICRUSt2) was employed to predict the abundance of bacterial functional genes in the KEGG-Level2 database (Figure 3a). Different treatments displayed higher expression abundances in metabolic pathways such as global and overview maps, cofactors and vitamins metabolism, and amino acid metabolism. Conversely, they exhibited lower abundance in pathways related to drug resistance (antineoplastic), endocrine and metabolic diseases, transport and catabolism, as well as environmental adaptation. Compared to CK, energy metabolism cofactors, and vitamins metabolism in treatments OF, NC, and FC increased by 24–32% and 25–33%, respectively.

Fungi Functional Guild (FUNGuild) was employed to analyze the expression abundance of functional genes in soil fungal communities across various treatments (Figure 3b). The analysis of functional group structure identified saprophytic fungi as the dominant functional gene in soil fungi. Except for unknown and undefined saprotrophs, the abundance of “*Animal Pathogen-Endophyte-Lichen Parasite-Plant Pathogen-Wood Saprotroph*,” “*Endophyte-Litter Saprotroph-Soil Saprotroph-Undefined Saprotroph*,” and “*Plant Pathogen*” were relatively higher than other functional genes. Particularly, the abundance of “*Animal Pathogen-Dung Saprotroph-Endophyte-Epiphyte-Plant Saprotroph-Wood Saprotroph*” in the FC treatment was significantly higher than in other treatments.

### 3.5 Relationships between soil microbial communities and environmental factors

**Figure 4:**
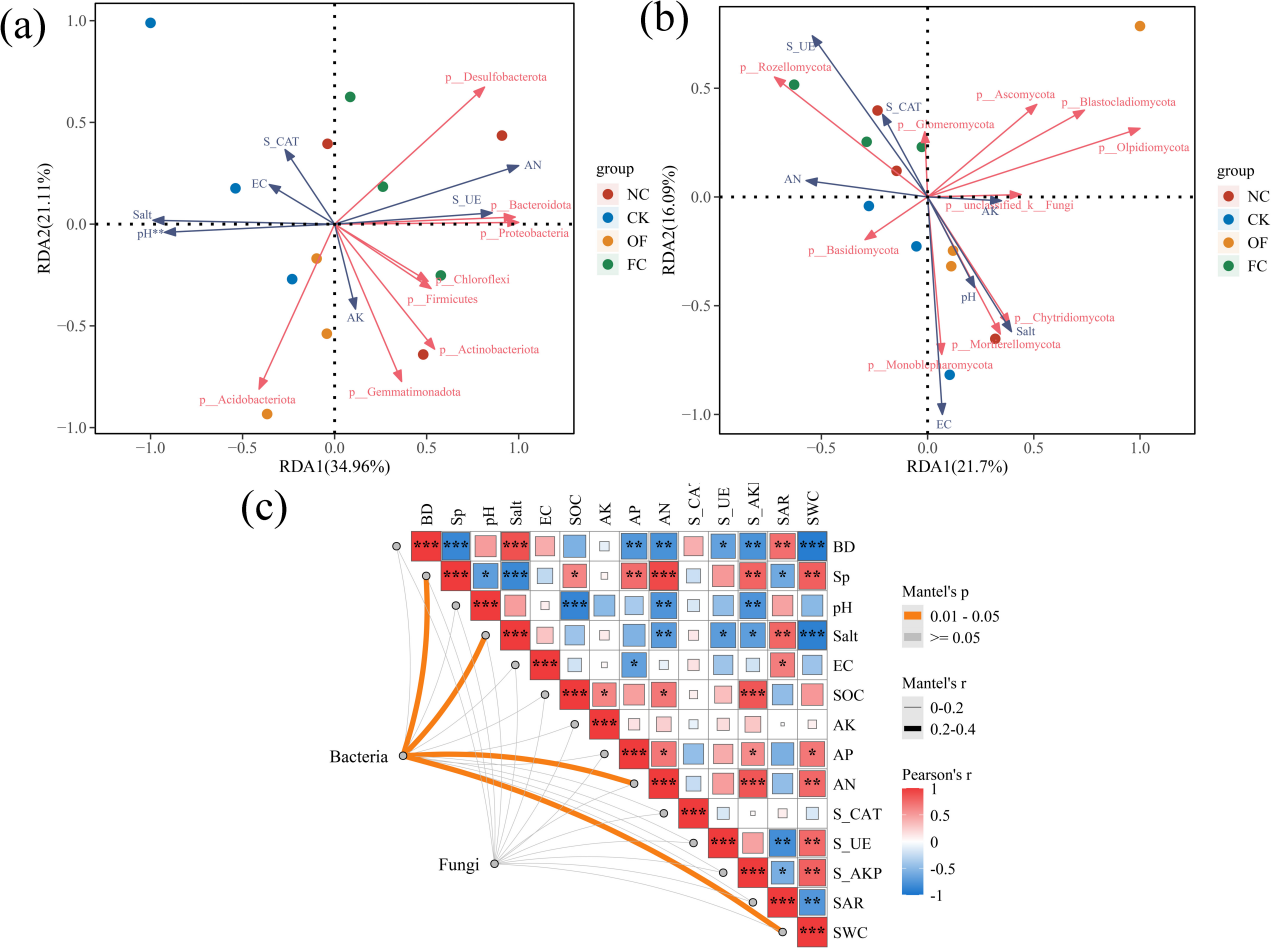
Redundancy analysis (RDA) of soil bacterial communities **(a)** and soil fungal communities **(b)** between different treatments and Mantel test analysis between selected soil properties and soil bacterial/fungal communities **(c)**. OF, bio-organic fertilizer; NC, nano carbon; FC, bio-organic fertilizer combined with nano carbon; CK, no amendments addition. Dots of different colors represent sample distributions for different treatments. The red arrows represent the different soil physicochemical factors, and the blue arrows represent the relevant dominant soil microbial communities. Corresponding significance levels, *p < 0.05, **p < 0.01, ***p < 0.001.

Redundancy analysis (RDA) was performed to elucidate the relationships between soil properties and the phyla-level composition of bacterial and fungal communities (Figure 4a). The RDA results revealed that the first and second axes explained 34.96% and 21.11% of the variation, respectively. *Proteobacteria, Bacteroidota, Desulfobacterota, Firmicutes*, and *Gemmatimonadota* showed significant negative correlations with soil salt, pH, and EC, while displaying significant positive correlations with soil available phosphorus, available nitrogen and urease activity (Figure 4a; Supplementary Figure S1). Table 2 indicated that soil available nitrogen, were the main factors influencing the distribution of soil bacterial communities (*p* < 0.05). The RDA results for fungal phyla capture a total explanation of 37.74% of fungal community variation (Figure 4b), reflecting the association between fungal phyla and soil environmental factors. The correlation heatmap revealed significant negative correlations of *Rozellomycota* with soil pH, while *Mortierellomycota* and *Chytridiomycota* exhibited positive correlations with soil pH (Supplementary Figure S2). In the ordination plots, samples from the FC treatment showed the most distinct separation from the control (CK), particularly for bacteria. Soil salinity (EC) and pH were the primary environmental drivers for both communities, jointly influencing the separation along the first RDA axis. The bacterial community was strongly shaped by the synergy of these two factors, whereas the fungal community was predominantly correlated with pH.

**Table 2:** Redundancy analysis results of soil bacterial and fungi at phylum level.

**Bacteria**					**Fungi**				
**Variable**	**Explains (%)**	**Contribution (%)**	*r* ^2^	***P*** **-value**	**Variable**	**Explains (%)**	**Contribution (%)**	*r* ^2^	***P*** **-value**
AN	27.41	37.49	0.54	0.006^**^	EC	12.23	19.88	0.130	0.217
Salt	9.624	13.15	0.37	0.255	S-UE	10.50	17.06	0.110	0.287
AK	8.168	11.16	0.29	0.312	AK	7.32	11.90	0.046	0.585
pH	7.88	10.78	0.22	0.321	AN	6.20	10.08	0.028	0.708
S-CAT	9.73	13.31	0.01	0.206	Salt	9.69	15.75	0.007	0.368
S-UE	6.41	8.767	0.01	0.425	S-CAT	7.04	11.44	0.005	0.674
EC	3.88	5.31	0.01	0.698	pH	8.52	13.85	0.004	0.544

The EC, AP, AK, AN, S_CAT, S_UE, S_AKP and represent soil bulk density, soil porosity, soil electrical conductivity, organic carbon, available soil phosphorus, available soil potassium, available soil nitrogen, soil catalase, soil urease, and soil alkaline phosphatase, respectively. Corresponding significance levels, ^*^p < 0.05, ^**^p < 0.01, ^***^p < 0.001.

The Mantel test was performed to assess the correlation between microbial community composition at the taxonomy level (based on Bray-Curtis dissimilarity of OTU profiles) and the dissimilarity matrix of measured soil physicochemical properties (Figure 4c). The results indicated statistically significant correlations between these matrices. Mantel's *r* values ranged from 0 to 0.2, supporting the role of soil physicochemical properties in shaping microbial community assembly. Bacterial community structure showed significant positive correlations with soil porosity, salt content, pH, available nitrogen, and soil water content, reflecting environmental filtering on bacterial growth and nutrient acquisition. In contrast, fungal community composition was not significantly correlated with soil EC or soil organic carbon (SOC; *p* ≥ 0.05), highlighting the distinct, context-dependent responses of bacterial and fungal communities to environmental gradients.

## 4 Discussion

### 4.1 Response of soil physicochemical properties to bio-organic fertilizer and nano-carbon addition

In this study, the introduction of organic amendments significantly enhanced soil porosity, soil organic carbon (SOC), available phosphorus (AP), soil water content (SWC), and available nitrogen (AN), while concurrently reducing soil electrical conductivity (EC), pH, sodium Adsorption Ratio (SAR), and bulk density (BD; Table 1). Similar enhancements in soil nutrient availability were noted by Wu et al. (2023) with the use of bio-organic fertilizer. Our findings further reveal that organic amendments not only boost soil nutrients and air permeability but also diminish soil alkalinity stress. The decomposition of organic fertilizer generates organic acids, which contribute to a lower soil pH (Ni et al., 2023). Additionally, the release of Ca^2+^ during this process helps displace exchangeable Na^+^ in the soil, which is vital for reducing soil dispersion (Zhao et al., 2022). Indeed, our study demonstrates that the incorporation of nano-carbon into the bio-organic fertilizer amendment significantly reduced soil sodium adsorption ratio (SAR), providing direct evidence of its effectiveness in mitigating saline-alkali stress (Table 3). Elemental analysis revealed a high oxygen content (49.81%) in the nano-carbon, indicating a surface enriched with oxygen-containing functional groups such as carboxyl (-COOH) and hydroxyl (-OH; Table 4). These functional groups are likely primarily responsible for the material's notable cation exchange capacity (CEC) and strong affinity for Na^+^ adsorption (Yan et al., 2024). This provides a plausible physicochemical mechanism underlying the observed reduction in soil sodium adsorption ratio (SAR), contributing to the mitigation of saline-alkali stress. This interpretation is consistent with recent studies on engineered biochars, in which increased oxygen functionality has been associated with enhanced sodium retention and improved soil CEC (Bhattacharya et al., 2025).

**Table 3:** Soil physical and chemical properties of different treatments in 0–20 cm.

**Sample**	**OF**	**NC**	**FC**	**CK**
BD (g cm^−3)^	1.38 ± 0.01b	1.29 ± 0.03c	1.27 ± 0.02c	1.44 ± 0.01a
Sp (%)	45.48 ± 0.39c	56.40 ± 0.83a	53.40 ± 1.35b	44.55 ± 0.60c
pH	8.41 ± 0.06a	8.30 ± 0.08b	8.22 ± 0.04b	8.52 ± 0.07a
Salt (g kg^−1^)	1.84 ± 0.02a	1.67 ± 0.06b	1.63 ± 0.06b	1.96 ± 0.06a
EC (us cm^−1^)	352.67 ± 18.80a	377.67 ± 52.87a	333.00 ± 31.87a	418.67 ± 13.70a
SAR	3.43 ± 0.13ab	3.22 ± 0.20b	2.46 ± 0.02b	4.09 ± 0.33a
SWC (%)	19.43 ± 0.32b	21.63 ± 0.60a	22.38 ± 0.92a	18.14 ± 0.10b
SOC (g kg^−1^)	9.23 ± 0.25b	10.47 ± 0.21a	9.20 ± 0.29b	8.60 ± 0.82b
AP (mg kg^−1^)	25.75 ± 0.15ab	30.97 ± 3.17a	30.77 ± 2.94a	24.74 ± 0.89b
AK (mg kg^−1^)	253.00 ± 12.68a	256.33 ± 7.04a	240.67 ± 3.40a	245.00 ± 8.04a
AN (mg kg^−1^)	59.53 ± 3.53c	76.61 ± 0.90a	69.53 ± 1.05b	60.34 ± 0.98c
S_CAT (U g^−1^)	117.19 ± 0.34a	116.98 ± 0.66a	116.56 ± 0.91a	117.05 ± 0.98a
S_UE (U g^−1^)	209.66 ± 29.87ab	211.15 ± 9.93ab	243.45 ± 9.46a	169.89 ± 9.19b
S_AKP (U g^−1^)	396.95 ± 34.87bc	701.67 ± 20.95a	570.60 ± 104.32ab	342.26 ± 112.10c

OF, NC, FC, and CK represent treatments of bio-organic fertilizer addition, nano carbon addition, bio-organic fertilizer combined with nano carbon, and no amendment addition, respectively. The BD, Sp, EC, SAR, SWC, SOC, AP, AK, AN, S_CAT, S_UE, S_AKP and represent soil bulk density, soil porosity, soil electrical conductivity, sodium adsorption ratio, soil water content, organic carbon, available soil phosphorus, available soil potassium, available soil nitrogen, soil catalase, soil urease, and soil alkaline phosphatase, respectively. Different lowercase letters indicate significant differences between OF, NC, FC, and CK within the same stand (Duncan's multiple tests, *p* < 0.05). All data showed with mean value ± standard deviation (*n* = 3).

**Table 4:** Different material compositions.

**Material**	**OF**	**NC**
pH	8.20	3.40
EC (ms cm^−1^)	23.20	0.27
SOM (g kg^−1^)	515.00	–
N (g kg^−1^)	25.60	0.70
P_2_O_3_ (g kg^−1^)	22.40	–
K_2_O (g kg^−1^)	18.60	–
Component	–	C/O
Carboxyl (mg g^−1^)	–	–
Bulk density (g cm^−3^)	–	–
C (%)	–	43.12
O (%)	–	49.81
H (%)	–	2.82

Notably, the FC treatment exhibited a marked improvement in nutrient enhancement and salinity reduction compared to other treatments. Nano-structured amendments have been identified as synergists for fertilizers, offering particular benefits under challenging soil conditions (Kheir et al., 2018; Cao et al., 2021). The rise in SOC coincided with an increased abundance of *copiotrophic* bacterial taxa, such as *Firmicutes* and *Bacteroidota*, which are typically associated with the utilization of labile carbon substrates. This suggests that the combined amendment supplied readily available carbon sources, thereby stimulating microbial biomass production (Meng et al., 2024). Notably, PICRUSt2-based functional predictions revealed enhanced potential for metabolic pathways related to cellular biosynthesis and energy metabolism-including cofactor and vitamin metabolism, as well as amino acid metabolism (Figure 3). The coupling of a stimulated copiotroph-dominated microbial community with upregulated biosynthetic capacity supports a mechanism whereby amendment-derived carbon is rapidly assimilated into microbial biomass (Somenahally et al., 2023). Subsequent microbial turnover and the accumulation of microbial necromass represent key processes contributing to SOC stabilization, consistent with the framework of the soil microbial carbon pump (Yao et al., 2022; Zhang et al., 2023).

Furthermore, amendments treatment has higher enzyme activity than CK, including S-UE and S-AKP. This phenomenon is attributable not only to the rich organic substrate present in the material but also to the incorporation of beneficial microorganisms that facilitate the decomposition of soil nitrogen and phosphorus, thereby enhancing enzyme activity (Wu et al., 2023; Guan H. et al., 2023). Further studies have demonstrated that nanomaterial application enhances nutrient availability, improves soil structure, and increases water retention (Cao et al., 2023). The synergistic impact of nano-carbon and bio-organic fertilizer likely facilitates the decomposition of organic matter, release of humic acids, improvement in soil structure, and ion leaching, as noted in previous research (Ondrasek and Rengel, 2021; Chandel et al., 2022), emphasizing the viability of nanocarbon for enhancing saline soils.

### 4.2 Response of soil microbial diversity and communities structures to bio-organic fertilizer and nano-carbon addition

Soils host diverse microbial communities that are crucial for maintaining soil functions (Bardgett and Van der Putten, 2014; Zhang et al., 2021). Organic amendments significantly increased soil microbial diversity, as indicated by the Ace, Chao1, and Shannon indices, while reducing the Simpson index (Figure 1). These results are consistent with findings by (Li et al. 2023) and Somenahally et al. (2023). In saline-alkali soils, high osmolality inhibits most microorganisms, favoring those that are salt-tolerant or obligate, thereby reducing community diversity and nutrient turnover (Zhang et al., 2023). Modified materials enhance microbial nutrients and improve the soil environment, mitigating osmotic stress and promoting microbial diversity (Zhu et al., 2021; Yao et al., 2022). Higher Alpha diversity indicates that the root-zone soil bacterial community is healthier, which is not conducive to the proliferation of pathogenic bacteria and the subsequent development of plant diseases (Zhu et al., 2021). Fungal communities, in particular, exhibited a stronger response to environmental improvement, consistent with their higher tolerance to salt stress and environmental adaptability (Zhang et al., 2021). This suggests that additions of fertilizer and nano-carbon may disproportionately benefit fungal alpha diversity over bacterial diversity, aligning with studies that show organic mulching enhances fungal dominance, which is crucial for soil organic matter (SOM) stabilization and soil health (Wang et al., 2020).

The addition of exogenous organic materials can alter the composition and relative abundance of microbial communities (Lu et al., 2023). In our study, at the phylum level, *Proteobacteria, Chloroflexi, and Actinobacteriota* were the dominant bacterial groups across various treatments, while *Desulfobacterota* and *Firmicutes* showed higher abundance in the FC treatment (Figure 2a). This observation is in line with previous research (Wang et al., 2020), which suggests that *Firmicutes* thrive in carbon-rich environments and are stimulated by the influx of high-quality exogenous organic carbon (Zheng et al., 2020). Additionally, *Firmicutes*, known for their ability to fix nitrogen, may increase plant susceptibility to diseases (Lu et al., 2023). They are also oligotrophic, adept at utilizing recalcitrant carbon sources, and secrete β*-glucosidase* and *xylanase* to decompose plant residues (Li et al., 2019). The abundance of *Firmicutes* in the FC treatment was significantly higher than that of the control not only because of the abundance of carbon metabolic substrates in the exogenous material, but also because the bioorganic fertilizer contained Bacillus and other genera of beneficial bacteria in the *Firmicutes* (Wang et al., 2020; Lee et al., 2025). Bacillus is a genus of beneficial bacteria in the Firmicu-tes, and studies have shown that Bacillus can have the functions of phosphorus and potas-sium decomposition and enhance soil metabolic functions (Lu et al., 2023). Furthermore, Desulfobacterota, part of the Deltaproteobacteria, plays a significant role in sulfur and nutrient cycling (Zhang et al., 2015). Fertilization in paddy fields may increase soil sulfate content, with Desulfobacterota converting sulfate to elemental sulfur or H_2_S, which can lower soil sulfate levels, while an optimal level of H_2_S can promote root growth (Jia et al., 2023). The enhanced presence of Firmicutes in the FC treatment could result from the elevated levels of organic materials, which increase soil sulfate content, leading to a reduction in soil pH due to the conversion of sulfate into H_2_S (Guan Y. et al., 2023).

Regarding fungal community distribution, dominant groups include *Ascomycota, Basidiomycota, Mortierellomycota, Zygomycota*, and *Glomeromycota* (Fernando et al., 2023). In the OF and FC treatments, *Ascomycota* exhibited significantly higher relative abundance compared to other treatments (Figure 2b). Existing research shows that *Ascomycota* and *Basidiomycota* serve as major decomposers of organic matter across various soil types due to their distinct nutritional niches. In our study, the pronounced relative abundance of *Ascomycota* in the OF and FC treatments can be attributed to the infusion of high-quality organic materials, as demonstrated by previous studies (Wang et al., 2020). Moreover, Mortierellomycota emerges as a potential biomarker for the NC treatment, capable of forming mycorrhizal symbiosis with plant roots, which facilitates the uptake of essential nutrients such as phosphorus and nitrogen, thereby enhancing plant growth and yield (Tarin et al., 2021; Yang et al., 2023). Additionally, Mortierellomycota contributes to soil aggregation and stability through the production of hyphae and secretion of extracellular compounds, improving water infiltration and retention (Iqbal et al., 2022).

Functional gene classification revealed only minor variations in bacterial metabolic pathways between different treatments, suggesting that short-term environmental changes have minimal impact on soil bacterial functions (Figure 3). However, organic treatments were associated with heightened expression of genes involved in energy and vitamin metabolism, likely bolstering bacterial stress resistance (Lin et al., 2021). FUNGuild analysis pointed to a dominance of saprotrophic fungi, consistent with earlier research (Figure 3; Liu et al., 2022; Liang et al., 2023), which plays a vital role in litter decomposition and maintaining soil ecology (Cao et al., 2023). Further studies should investigate the long-term responses of microbial communities to changes in soil water content and fertilization practices.

### 4.3 Organic amendments drive soil microbial community responses and influence soil properties

Soil bulk density, pH, EC, organic carbon, urease, and alkaline phosphatase were identified as primary factors influencing the abundance of bacterial communities at the phylum level (Figures 4a, b). Among these, bulk density, pH, and EC were found to negatively correlate with major bacterial groups, echoing findings from previous research (Wang et al., 2020). In saline soils, pH and salinity stress microbial communities, thereby altering the substrate composition (Peng et al., 2023). Organic amendments have been shown to reduce pH and bulk density while enhancing organic carbon content and the availability of nutrients, which is reflected in the increased abundance of dominant bacterial communities. Redundancy analysis (RDA) underscored the significant impact of soil porosity and bulk density on bacterial communities, as observed in earlier studies (Table 2; Artyszak and Gozdowski, 2020).

The amendment-induced shifts in soil physicochemical conditions likely established a strong environmental filter, playing a pivotal role in shaping microbial community assembly (Li et al., 2025). Multivariate analyses (Mantel test) revealed significant correlations between reduced salinity (Figure 4c), altered nutrient status, and bacterial community restructuring. This supports the growing understanding that carbon-based nanomaterials can influence soil microbial composition (Das et al., 2025). The robust and widespread associations between bacterial community structure and amended soil properties suggest that bacteria respond directly and rapidly to such abiotic changes (Xia et al., 2024). In contrast, the fungal community displayed a divergent response pattern, evidenced by the absence of significant correlations with key amended factors such as soil organic carbon (Figure 4c). The NMDS points of the fungal community are more dispersed, indicating that the heterogeneity of the fungal community structure among treatments is higher than that of the bacterial community (Figure 2). This may be related to the stronger specificity of the fungal response to abiotic factors, implies that fungal assembly in this system may be governed by more complex mechanisms, including microhabitat heterogeneity, biotic interactions, or delayed responses to changes in carbon availability (Tu et al., 2024). The apparent decoupling between bacterial and fungal responses highlights the necessity of examining these microbial groups independently to achieve a comprehensive assessment of amendment effects on soil ecological dynamics.

Finally, it should be noted that the functional interpretations regarding microbial metabolism in this study are derived from PICRUSt2 predictions, and should therefore be interpreted with caution as inferred hypotheses rather than confirmed mechanisms. Future efforts should therefore focus on direct experimental validation, such as through metagenomic sequencing, quantitative PCR (qPCR) of key functional genes, or assays of enzyme activities, to substantiate these predicted metabolic pathways. Furthermore, to comprehensively evaluate the agricultural potential of this synergistic amendment, subsequent research should employ pot or field plot trials designed to systematically measure crop growth, yield parameters, and rhizosphere microbial dynamics.

## 5 Conclusion

The synergistic application of nano-carbon and bio-organic fertilizer (FC) most effectively improved coastal saline soil by reducing bulk density, salinity, pH, and SAR, while enhancing porosity, water retention, and nutrient availability. This created a favorable habitat that significantly increased bacterial diversity and selectively enriched functional taxa (e.g., Firmicutes, Desulfobacterota), with soil salinity and pH identified as key drivers. In contrast, fungal communities showed distinct, non-correlated responses, suggesting different assembly mechanisms. The predicted functional shifts require direct validation. Ultimately, FC amendment appears to promote soil health primarily by rapidly ameliorating the physicochemical environment to directionally shape the bacterial community, highlighting its potential as a strategy for the initial restoration of saline-alkali ecosystems. Future work should focus on functional verification and agronomic outcome assessments.

## Data Availability

The data presented in the study are deposited in the NCBI Sequence Read Archive repository, accession number PRJNA1419807.

## Supplementary material

Supplementary Figure S1 Correlation analysis between the top 15 dominant bacterial groups with relative abundance and soil environmental factors at the phylum level. Corresponding significance levels, ^*^*p* < 0.05, ^**^*p* < 0.01, ^***^*p* < 0.001.

Supplementary Figure S2 Correlation analysis between the top 13 dominant flora with relative abundance and soil environmental factors at phylum level. Corresponding significance levels, ^*^*p* < 0.05, ^**^*p* < 0.01, ^***^*p* < 0.001.

Supplementary Figure S3 ANOSIM analysis box plot. The X-axis represents the distance values within or Between groups. The boxes corresponding to Between represent the distance values of differences between groups, and the remaining boxes represent the distance values of differences within groups. The Y-axis scale indicates the magnitude of the distance value. Corresponding significance levels, ^*^*p* < 0.05, ^**^*p* < 0.01, ^***^*p* < 0.001.

## Appendix

**Table A1:** Sequencing information of each sample.

**Sample**	**Valid reads**	**Average length**	**Total bases**	**Q30**	**Q20**
OF1	58,580	238.294	13,959,250	98.2783	99.2704
OF2	63,158	240.472	15,187,738	98.4349	99.3404
OF3	74,149	239.808	17,781,514	98.4079	99.3462
NC1	61,108	236.197	14,433,553	98.4212	99.3371
NC2	66,673	244.177	16,280,025	98.3151	99.3095
NC3	62,350	230.32	14,360,475	98.8381	99.4926
FC1	68,104	228.091	15,533,942	98.9717	99.5918
FC2	68,996	220.031	15,181,270	99.2088	99.6965
FC3	68,429	215.712	14,760,986	99.1437	99.6441
CK1	56,415	282.773	15,952,653	97.6732	99.1073
CK2	49,505	235.502	11,658,551	98.6254	99.4341
CK3	57,765	242.548	14,010,783	98.4424	99.366

Q30, the percentage of bases with a base recognition accuracy rate of over 99.9%; Q20, the percentage of bases with a base recognition accuracy rate of over 99%.

**Table A2:** ANOSIM analysis result.

**Method**	**Statistic**	***P*-value**	**Permutation_number**
ANOSIM	0.259259	0.042^*^	999

Corresponding significance levels, ^*^*p* < 0.05, ^**^*p* < 0.01, ^***^*p* < 0.001.
