# Supplementary figures and images for: Synergistic nano-bioorganic amendments enhance soil properties and microbial structure in coastal saline soils
Source: Front Microbiol. 2026 Mar 9;17:1720097. doi: 10.3389/fmicb.2026.1720097 (PMC13006664; doi:10.3389/fmicb.2026.1720097)

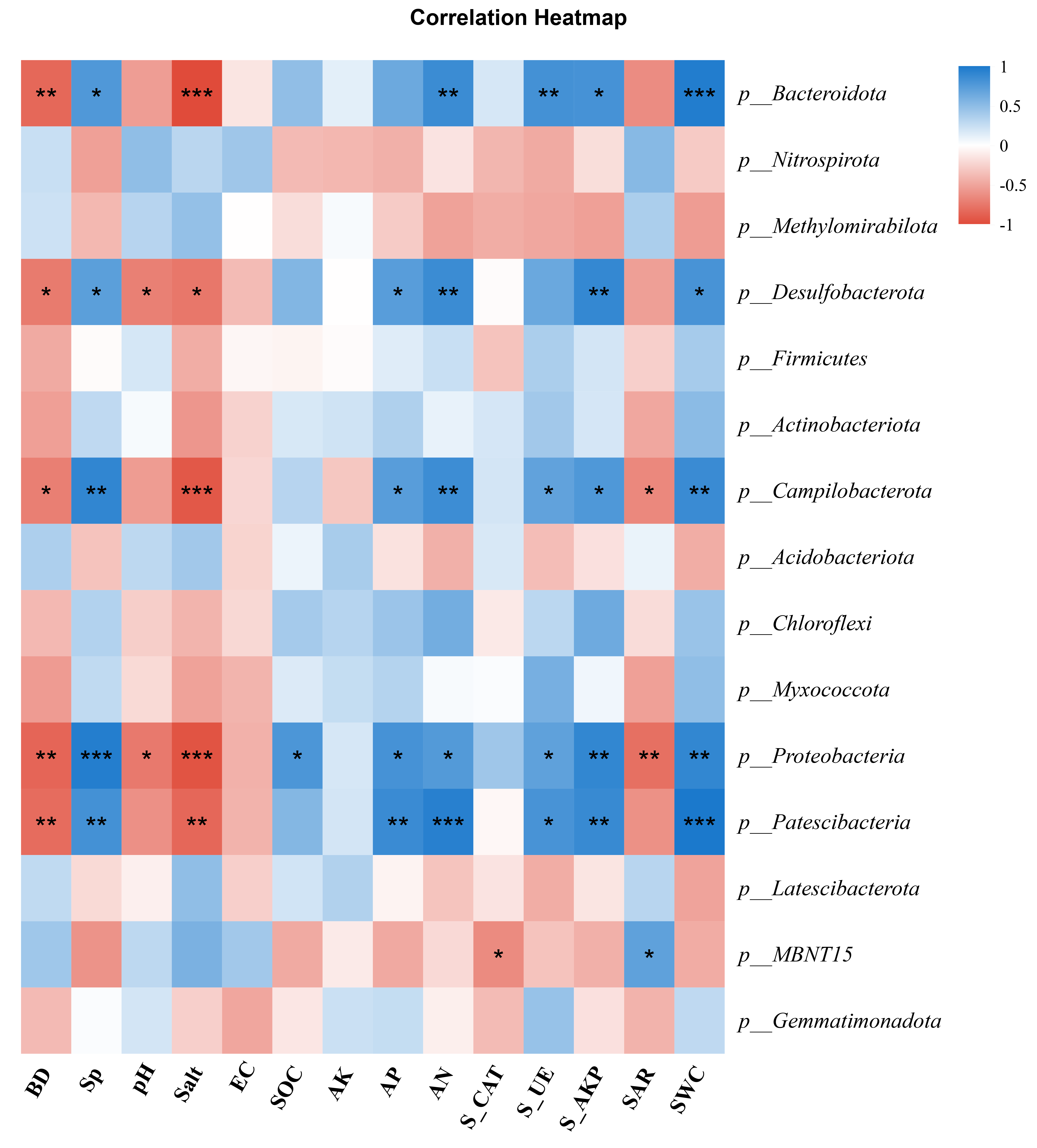

Supplement: Supplementary Figure S1 — Correlation analysis between the top 15 dominant bacterial groups with relative abundance and soil environmental factors at the phylum level. Corresponding significance levels, *p < 0.05, **p < 0.01, ***p < 0.001. [file Image_1.tiff]

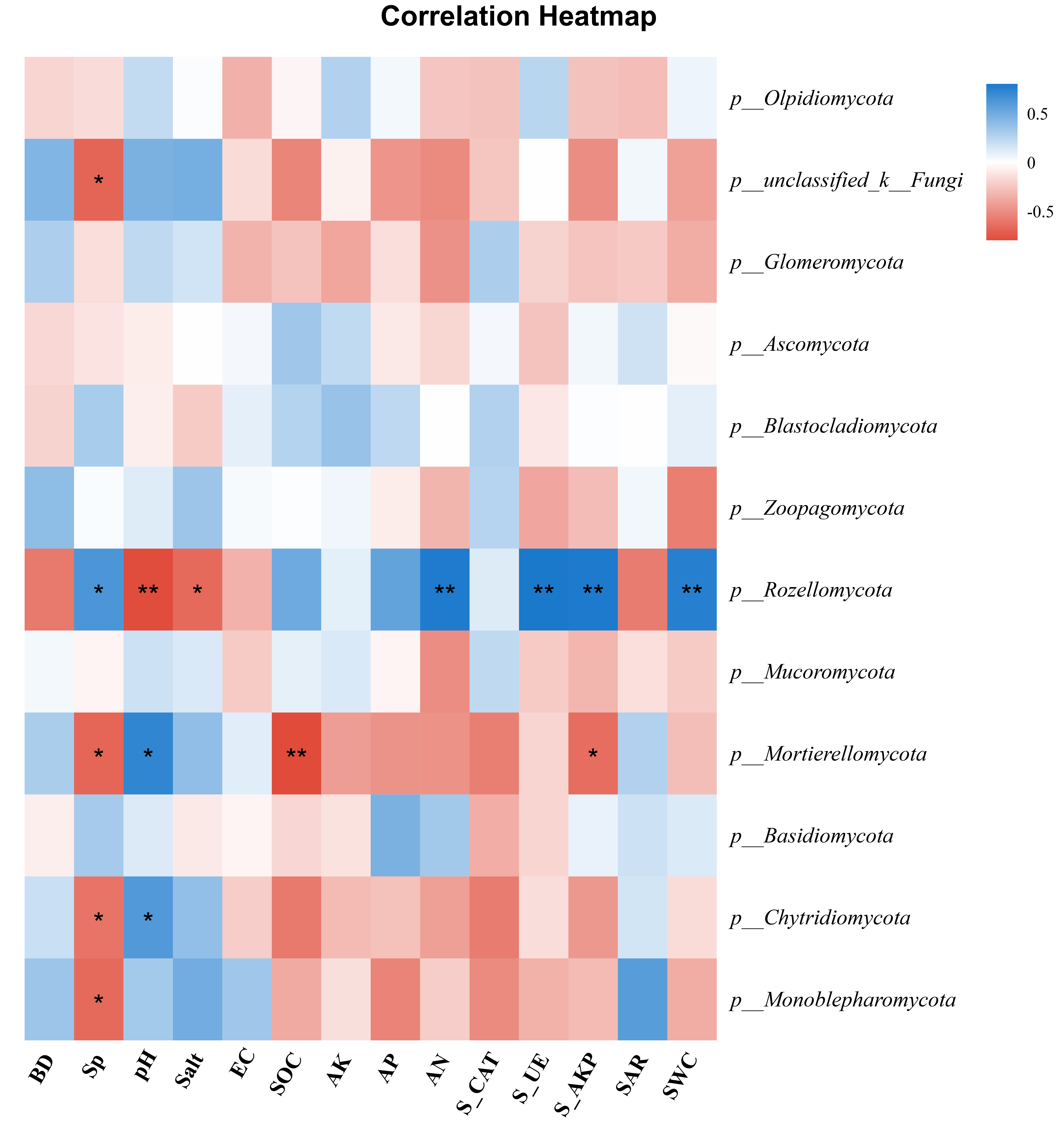

Supplement: Supplementary Figure S2 — Correlation analysis between the top 13 dominant flora with relative abundance and soil environmental factors at phylum level. Corresponding significance levels, *p < 0.05, **p < 0.01, ***p < 0.001. [file Image_2.tiff]
